# Evaluating the cost of adult voluntary medical male circumcision in a mixed (surgical and PrePex) site compared to a hypothetical PrePex-only site in South Africa

**DOI:** 10.3402/gha.v8.29116

**Published:** 2015-12-15

**Authors:** Hae-Young Kim, Limakatso Lebina, Minja Milovanovic, Noah Taruberekera, David W. Dowdy, Neil A. Martinson

**Affiliations:** 1 Department of Epidemiology, Johns Hopkins Bloomberg School of Public Health, Baltimore, MD, USA; 2 Perinatal HIV Research Unit, Faculty of Health Sciences, University of the Witwatersrand, Johannesburg, South Africa; 3 Population Services International, Johannesburg, South Africa; 4 Center for Tuberculosis Research, Johns Hopkins University School of Medicine, Baltimore, MD, USA

**Keywords:** male circumcision, cost analysis, PrePex, HIV prevention, South Africa

## Abstract

**Background:**

Several circumcision devices have been evaluated for a safe and simplified male circumcision among adults. The PrePex device was prequalified for voluntary male medical circumcision (VMMC) in May 2013 by the World Health Organization and is expected to simplify the procedure safely while reducing cost. South Africa is scaling up VMMC.

**Objective:**

To evaluate the overall unit cost of VMMC at a mixed site vs. a hypothetical PrePex-only site in South Africa.

**Design:**

We evaluated the overall unit cost of VMMC at a mixed site where PrePex VMMC procedure was added to routine forceps-guided scalpel-based VMMC in Soweto, South Africa. We abstracted costs and then modeled these costs for a hypothetical PrePex-only site, at which 9,600 PrePex circumcisions per year could be done. We examined cost drivers and modeled costs, varying the price of the PrePex device. The healthcare system perspective was used.

**Results:**

In both sites, the main contributors of cost were personnel and consumables. If 10% of all VMMC were by PrePex at the mixed site, the overall costs of the surgical method and PrePex were similar – US$59.62 and $59.53, respectively. At the hypothetical PrePex-only site, the unit cost was US$51.10 with PrePex circumcisions having markedly lower personnel and biohazardous waste management costs. In sensitivity analysis with the cost of PrePex kit reduced to US$10 and $2, the cost of VMMC was further reduced.

**Conclusions:**

Adding PrePex to an existing site did not necessarily reduce the overall costs of VMMC. However, starting a new PrePex-only site is feasible and may significantly reduce the overall cost by lowering both personnel and capital costs, thus being cost-effective in the long term. Achieving a lower cost for PrePex will be an important contributor to the scale-up of VMMC.

In 2007, the World Health Organization (WHO) and the Joint United Nations Program on HIV/AIDS (UNAIDS), consequent to the findings of three clinical trials (1–3), recommended the inclusion of voluntary medical male circumcision (VMMC) as an HIV prevention strategy (4). Fourteen priority countries in sub-Saharan Africa were identified for the scale-up of VMMC and together agreed to circumcise 28 million men by 2025 with the aim of averting three million HIV infections (4).

South Africa set its own targets of circumcising 4.3 million men by 2016, but by March 2014 had only circumcised 1.4 million (5, 6). To make more rapid progress toward these targets, interventions to facilitate rapid scale-up of VMMC are urgently needed. Device-based circumcision has been touted as a potential contributor to such progress, and efforts to test candidate devices are underway. The PrePex male circumcision device was preapproved for male circumcision by the WHO in June 2013 and is currently undergoing field testing including surveillance of adverse events in South Africa and other countries (7).

Several studies have assessed PrePex circumcision, suggesting it is more cost-effective and efficient per unit used; yet others suggest that cost saving is unlikely, especially when procedural costs (site, waste disposal, staff, and equipment) are considered (8, 9). In this paper, we measured the costs of VMMC at a mixed site where both forceps-guided male circumcision (FGMC) and PrePex circumcisions were offered. Applying these costs, we then estimated costs of a hypothetical PrePex-only circumcision clinic.

## Methods

### Study population and setting

Costs for FGMC were obtained from the high-volume VMMC clinic at Chris Hani Baragwanath Academic Hospital in Soweto, South Africa, at which PrePex circumcision was introduced in June 2014 after 4 years of FGMC. The time and personnel required to perform FGMC were obtained from directly observing 20 patients at the clinic. The time and personnel required to perform PrePex circumcision were collected as a part of a WHO-recommended pilot study across three clinics: two free-of-charge VMMC clinics (Witbank Hospital and Tsakane Clinic) and one fee-for service (Zuzimpilo Clinic). At these sites, males requesting circumcision were offered PrePex if they were willing and eligible; otherwise the forceps-guided method was used. We limited our analysis to the total of 348 adult males (age ≥18 years) in whom safety and acceptability of PrePex had been established (10). Individual consent for circumcision was obtained from all those who were circumcised.

The mixed site had the following personnel: two deputy program directors, one medical officer, two clinical associates, two professional nurses, six enrolled nurses, five counselors, two data capturers, three filed workers, three receptionists, one social mobilization coordinator, one social worker, and one general assistant. The following procedure was conducted for FGMC. After HIV testing and counseling were provided by a counselor, an enrolled nurse conducted a brief physical examination to check the client's eligibility for circumcision. During the surgical operation, local anesthesia was performed by an enrolled nurse. A doctor or a clinical associate initiated the removal of the foreskin and sutures, which was usually completed by an enrolled nurse. After surgery, each client was transferred to the recovery room where he was provided with post-surgery counseling and scheduled for follow-up visits.

The PrePex-only clinic was assumed to have the minimal number of personnel for operation and a floor area of 112 m^2^ based on the floor plan of a circumcision clinic, including: reception, waiting room, four counseling rooms, a consultation room, procedure room, recovery room, and toilets. We assumed that such space would provide the capacity to do 20 PrePex circumcisions and 20 removals each working day at the maximum capacity.

### Data collection

Unit costs for both procedures were ascertained using an ‘ingredients’ (bottom-up) approach from the healthcare perspective, with the costs of individual components added to calculate a unit cost for each circumcision. All analyses were done by the Decision-Makers’ Program Planning Tool (DMPPT) developed by the UNAIDS in 2010 (11). We categorized costs into direct and indirect costs as referred in DMPPT. The direct cost per circumcision included costs of consumables, non-consumable supplies, personnel, training, and waste management. The costs of consumables and equipment required for both FGMC and PrePex circumcisions were abstracted from the invoices of ordered products or quotations. For the items purchased in prior years, costs were inflated using the Gross Domestic Product deflator for South Africa compared to the reference year of 2005 (12). We used the conversion rate of 10.62 ZAR to 1 dollar. Duration of the circumcision procedure was measured from a patient's entering into the theatre or the consulting room to exiting the room after having FGMC or having the PrePex device applied. Twenty surgical VMMC procedures were directly observed. The time contributed by each healthcare worker in doing either FGMC or PrePex circumcision was recorded, and current annual salaries of staff directly involved in circumcision were used to calculate the personnel cost per circumcision. FGMC was normally performed by a medical officer or a clinical associate, paired with one enrolled nurse. We included costs of HIV counseling and testing but no other activities at the circumcision site.

PrePex training required one medical officer and five enrolled nurses to spend 5 days each, requiring 15 applications and 10 removals to be done under the direct supervision of a PrePex master trainer. We included costs of training two professional nurses and two enrolled nurses for 5 days for the hypothetical PrePex-only site. Counselors were considered to receive 1 day training at each site. Health workers in the circumcision program had a separate training on a model for optimizing the volume and efficiency (MOVE). For FGMC, 2 days of MOVE training were conducted. Training was assumed to occur annually at the beginning of the program implementation.

At the mixed site, the number of circumcisions was 70% lower outside of winter than during the three winter months; thus, we assumed that a PrePex-only site could operate at this full capacity for 3 months per year and would operate at 30% capacity (six circumcisions and six removals per day) during the other 9 months. Based on the data from PrePex circumcisions, we assumed that 1.3% of patients attending the PrePex-only site would be transferred to a nearby mixed site due to ineligibility to perform the PrePex procedure, or other complications of the procedure requiring surgical revision. The cost for transportation was not included. For both FGMC and PrePex circumcision, the monthly volume of biohazardous waste generated per circumcision was estimated to calculate the unit cost of waste removal. For indirect costs, we included costs for capital, maintenance and utility, support personnel, and management and supervision. Items used more than 1 year such as equipment and furniture were considered as capital and annualized using an expected life of 2 to 5 years. We assumed that the mixed site could be operated using existing infrastructure, but a PrePex-only site in the community would require additional construction, the cost of which we estimated based on the quotation for the proposed floor plan, annualized over a 20-year estimated life expectancy.

### Sensitivity analyses

We performed one-way sensitivity analyses on all parameters including the time for FGMC or PrePex circumcision and the number of support personnel, and reported on the parameters that most influenced the cost estimates. For parameters with an appropriate range of data the 2.5th and 97.5th percentiles were reported to generate 95% uncertainty ranges. If limited data were available, we varied the base value of each parameter by ±50%. The ranges for model parameters are listed in Table 2. We further examined the effect of different PrePex kit prices at US$2 and $10 and the volume of demand. Because the demand for circumcision greatly varies depending on season with high volume in winter (100–120 per day) and low volume in non-winter (1–20 per day), we performed a scenario analysis in which we evaluated the unit cost in winter (June–August) and non-winter (September–May) separately.

## Results

### Costs of circumcision

For a single FGMC, the average time taken by an enrolled nurse and a medical officer or a clinical associate was 31.1±5.4 min and 5.3±1.0 min, respectively. Of 348 PrePex circumcisions, 67 PrePex applications were done by one operator and one assistant at an average time of 7.9±4.3 min. Assuming that 10% of all circumcisions done at the mixed site were PrePex applications, the unit costs of the FGMC and PrePex procedure were similar at US$59.62 and $59.53, respectively, resulting in an average per-circumcision cost of US$59.61. In the hypothetical PrePex-only site, the unit cost of PrePex circumcision was lower (US$51.10), and the average unit cost per circumcision, including 1.3% of men requiring FGMC to resolve an adverse event, was US$51.21 (Table 1).

**Table 1:** Unit cost for forceps-guided male circumcision and PrePex male circumcision in a mixed site and a hypothetical PrePex-only site in South Africa

	Mixed site				Hypothetical PrePex-only site	
		
Cost category	Surgery		PrePex		PrePex	
Direct costs						
Consumables	$18.77	32%	$24.33	41%	$24.33	48%
Non-consumable supplies	$0.04	0%	$0.04	0%	$0.04	0%
Waste management	$4.01	7%	$1.11	2%	$1.11	2%
Personnel	$8.00	13%	$5.27	9%	$5.27	10%
Training	$1.05	2%	$1.05	2%	$0.83	2%
Subtotal	$31.86	54%	$31.80	53%	$31.58	62%
Indirect costs						
Capital	$1.58	3%	$1.58	3%	$1.97	4%
Maintenance and utility	$8.23	14%	$8.23	14%	$6.58	13%
Support personnel	$13.81	23%	$13.81	23%	$7.73	15%
Management and supervision	$3.93	7%	$3.93	7%	$3.15	6%
Subtotal	$27.56	46%	$27.56	47%	$19.42	38%
Total unit cost	$59.42		$58.82		$51.00	
Total unit cost weighted with complications	$59.62		$59.53		$51.10	
Average unit cost weighted with complications	$59.61				$51.21	

Consumables (US$24.33) contributed 48% of the total cost in the hypothetical PrePex-only site, versus 31% (US$18.77) of the FGMC in the observed mixed site. Waste management costs were higher for FGMC (US$4.01) compared to PrePex (US$1.11). Direct personnel costs were US$2.73 lower in the PrePex circumcision compared to FGMC. Support personnel costs contributed 23% of the total cost in the mixed site, compared to 15% in the PrePex-only site. Overall, the cost of providing 10,000 circumcisions in a mixed site with a PrePex kit priced at US$20 was US$596,110, compared to US$513,193 at the hypothetical PrePex-only site. Changing the proportion of PrePex circumcisions in the mixed site did not substantively affect the results.

### Sensitivity analyses

**Fig. 1:**
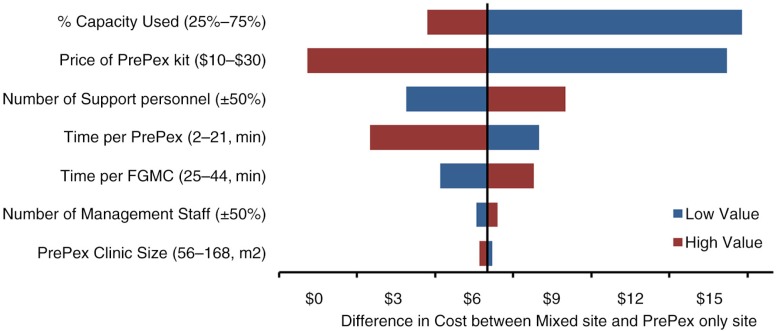
One-Way Sensitivity Analysis. Shown are all parameters that, when varied across the ranges shown, changed the estimated difference in cost of circumcision (mixed site minus PrePex-only site).

Key parameters and sensitivity ranges are shown in Table 2. Figure 1 shows the difference in cost between the mixed site and the hypothetical PrePex-only site upon variation of each key parameter across its sensitivity range. The biggest driver of this difference was the percentage of capacity used, followed by the PrePex kit price. At 25% of full capacity, the unit cost at the mixed site was US$18.20 higher than at the PrePex-only site. Having 50% less support personnel at both sites reduced the difference in the cost to US$5.30. At the 97.5th percentile of observed PrePex application time (21 min), the unit cost at the mixed site was still US$3.90 greater than at the PrePex-only site. Varying the number of management staff or the size of PrePex clinic had only minimal effect.

**Table 2:** The range of key parameters in the cost model

	Mixed site		Hypothetical PrePex-only site	
		
	Baseline	Range (2.5th–97.5th percentiles)	Baseline	Range (2.5th–97.5th percentiles)
**Personnel**				
Time per FGMC (min)				
1 Enrolled nurse	31.1±5.4	21, 37	N/A	
1 Medical officer or 1 clinical associate	5.3±1.0	4, 7		
Time for PrePex circumcision (min)				
1 Enrolled nurse and 1 professional nurse	7.9±4.3	2, 21	7.9	2, 21
	Baseline	(−50%, +50%)	Baseline	(−50%, +50%)
Support personnel				
Number of full-time support personnel	14	7, 21	4	2, 6
Number of management staff	2	1, 3	1	0, 2
Capital				
Facilities size (m^2^)	N/A		112	56, 168
Patient volume (capacity)				
Annual utilization of capacity(%)	49.5	24.8, 74.3	49.5	24.8, 74.3

### Cost of PrePex kit

**Fig. 2:**
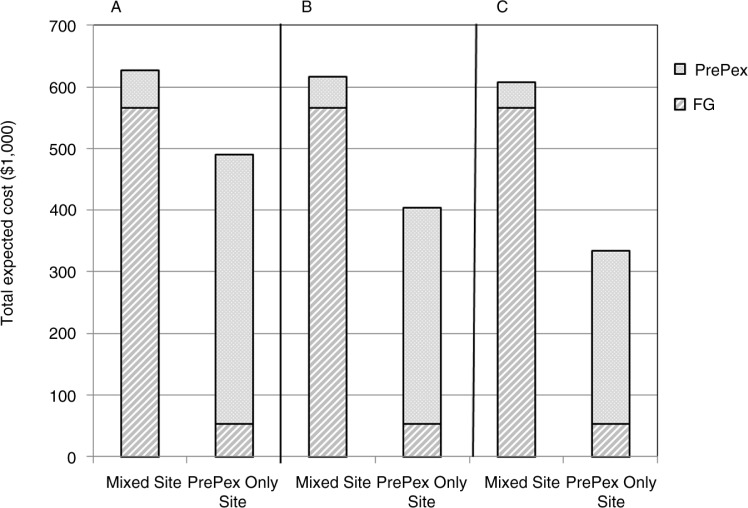
Total expected cost for 10,000 male circumcision at the mixed site and a PrePex Only site. Costs were modeled for different costs of PrePex kit at A) PrePex at $20 B) PrePex at $10, and C) PrePex at $2.

In both sites, the price of PrePex kit was a primary driver for the PrePex circumcision. If the unit cost of a PrePex kit was reduced to US$10, the average unit cost of PrePex circumcision would be reduced to US$49.53 in the mixed site and $41.21 in the PrePex-only site. With PrePex kits available at a price of US$2 per kit, the cost of circumcision would be further reduced to US$31.21 in the hypothetical PrePex-only site. The total expected costs of performing 10,000 male circumcisions at the mixed site or at the PrePex-only site with different PrePex device prices are shown in Fig. 2.

### Seasonal effect

The unit cost in winter in the mixed site was US$44.96 and $44.36 for FGMC and PrePex circumcisions, respectively. During non-winter months, the unit cost nearly doubled to US$73.82 and $73.23, respectively. The main driver for higher costs in non-winter time was suboptimal use of facilities and personnel due to lower volume of patients (Table 1).

## Discussion

In this analysis, we show that adding the PrePex procedure to an existing site did not markedly reduce the overall costs of VMMC. However, establishing a PrePex-only site with minimal work force appeared to reduce the overall cost by reducing the need for more expensive personnel. The mixed site facilitates outreach to more people by having higher capacity and conducting circumcision for people who fail or are ineligible for PrePex circumcision. However, having PrePex-only clinics could be more accessible to clients and require fewer personnel, and thus could be an alternative approach to rapidly scale-up VMMC. These satellite PrePex clinics can coordinate with a central mixed site to refer ineligible or complicated cases.

Training is a potential limitation to PrePex scale-up. In South Africa, there are few certified trainers; thus, healthcare providers in suburban or rural areas must often travel to training centers that are hours away and stay for 1–2 weeks. Although training costs accounted for only 2% of the total costs in this study and do not depend on the volume of trainees, having more trainees for one session may reduce other related costs such as transportation. Also, it is important to have more certified trainers accessible to healthcare providers in rural areas to scale-up PrePex circumcision.

In both sites, seasonality was a major limiting factor to reaching full capacity, leading to the increases in indirect costs. In the non-winter seasons, the average volume of patients at clinic was about 30% of full capacity, and the unit cost at the hypothetical PrePex-only site was significantly lower than at the mixed site. A recent case study in Tanzania has reported that overcoming seasonality is possible through a year-long demand generation campaign (13). Despite a stated target of circumcising 80% of all adult men in South Africa by 2015, only 40.6 and 46.4% of males aged 15 years or older had been circumcised by 2008 and 2012, respectively (14). A recent study in Kenya showed that 99% of participants who received PrePex VMMC would recommend it to male friends and family members (10), and the adverse rates from several reports were very low. These findings strongly suggest that PrePex VMMC can be an alternative strategy to increase the uptake of male circumcision.

Our evaluation has several limitations. We included costs from the healthcare perspective but did not include costs incurred by patients such as transportation fees or opportunity costs. The unit cost reflects the circumcision procedure but does not include other costs such as HIV counseling in the planning and budgeting for circumcision programs, these costs should be considered. Finally, the cost and feasibility of constructing a PrePex-only site can vary greatly but our analysis suggests that a PrePex-only site may not only be cost-effective in the long term but could also serve as a primary care clinic once the demand for circumcision slows down.

Further research is needed to maximize the capacity to scale-up VMMC and the effectiveness of PrePex-focused clinics. This empirical costing analysis suggests that fostering a PrePex-only model of care delivery (with a minimum price tag for PrePex) may improve cost-effectiveness, which in turn could be an important contributor to the scale-up of VMMC.
